# 7q35 Microdeletion and 15q13.3 and Xp22.33 Microduplications in a Patient with Severe Myoclonic Epilepsy, Microcephaly, Dysmorphisms, Severe Psychomotor Delay and Intellectual Disability

**DOI:** 10.3390/genes11050525

**Published:** 2020-05-08

**Authors:** Francesco Paduano, Emma Colao, Sara Loddo, Valeria Orlando, Francesco Trapasso, Antonio Novelli, Nicola Perrotti, Rodolfo Iuliano

**Affiliations:** 1 Medical Genetics Unit, University “Magna Graecia”, 88100 Catanzaro, Italy; francesco.paduano@tecnologicasrl.com (F.P.); colaoemma@gmail.com (E.C.); trapasso@unicz.it (F.T.); perrotti@unicz.it (N.P.); 2 Tecnologica Research Institute and Marrelli Health, Biomedical Section, Stem Cells Unit, 88900 Crotone, Italy; 3 Department of Experimental and Clinical Medicine, University Magna Graecia of Catanzaro, Campus S. Venuta, Viale Europa, Località Germaneto, 88100 Catanzaro, Italy; 4 Medical Genetics Laboratory, Bambino Gesù Pediatric Hospital, IRCCS, 00165 Rome, Italy; sara.loddo@opbg.net (S.L.); valeria.orlando@opbg.net (V.O.); antonio.novelli@opbg.net (A.N.); 5 Department of Health Sciences, University “Magna Graecia”, 88100 Catanzaro, Italy

**Keywords:** copy number variants (CNVs), neurodevelopmental disorders, microduplication, microdeletion, *CNTNAP2*, *CHRNA7*, *CRLF2*

## Abstract

Copy number variations (CNVs) play a key role in the pathogenesis of several diseases, including a wide range of neurodevelopmental disorders. Here, we describe the detection of three CNVs simultaneously in a female patient with evidence of severe myoclonic epilepsy, microcephaly, hypertelorism, dimorphisms as well as severe psychomotor delay and intellectual disability. Array-CGH analysis revealed a ~240 kb microdeletion at the 7q35 inherited from her father, a ∼538 kb microduplication at the 15q13.3 region and a ∼178 kb microduplication at Xp22.33 region, both transmitted from her mother. The microdeletion in 7q35 was included within an intragenic region of the contactin associated protein-like 2 (*CNTNAP2*) gene, whereas the microduplications at 15q13.3 and Xp22.33 involved the cholinergic receptor nicotinic α 7 subunit (*CHRNA7*) and the cytokine receptor-like factor 2 (*CRLF2*) genes, respectively. Here, we describe a female patient harbouring three CNVs whose additive contribution could be responsible for her clinical phenotypes.

## 1. Introduction

Copy number variations (CNVs) are deletions or duplications of genomic DNA larger than 1 kilobase [1], which may affect dosage balance in genes within CNVs or disrupt the regulatory regions, resulting in variations of allelic expression or altered gene functions [2]. Recurrent CNVs can occur in the presence of low-copy repeats (LCRs), genomic sequences highly susceptible to rearrangements and known to be hotspots for CNVs formation [3,4].

CNVs may be inherited from a parent or arise from de novo in an individual [5]. CNVs can occur in healthy individuals as benign polymorphic variants [6] and therefore contributing to human genetic diversity [1]. However, they can cause Mendelian and complex multifactorial disease, including neurological disorders [6], with a crucial role in the pathogenesis of neurodevelopmental disorders such as schizophrenia [7,8], intellectual disability (ID) [9], and autism spectrum disorder (ASD) [10], as well as in epilepsy [8]. 

In 2008, the international schizophrenia consortium described subjects with schizophrenia having 7q35 loss CNVs involving *CNTNAP2* [7]. A deletion at 7q35 involving the same gene was observed in a patient with ASD and ID [11]. All these data underline the important role of this gene in the development of the nervous system.

Deletions in intron 1 of *CNTNAP2* (CNVs>80kb) have also been found in normal healthy subjects, as described in the database of genomic variants (DGV: http://dgv.tcag.ca/dgv/app/home). Thus, CNVs are present not only patients with disease but can also be present in the general population.

One of the recurrent CNV regions associated with the development of several neuropsychiatric diseases is 15q13.3 [12,13]. Duplications at 15q13.3 involving *CHRNA7* have been associated with numerous neurological and neuropsychiatric phenotypes including ID [14], epilepsy [15], attention deficit hyperactivity disorder (ADHD) [16], ASD [14,17], language impairment [17,18], BPD [14,19], OCD [17,20], adult-onset schizophrenia (AOS) [21], developmental delay [17], behavioural disorders [17] as well as GTS [20]. Therefore, it has been shown that patients having duplication at 15q13.3 exhibit a variety of neuropsychiatric disorders and possess an incomplete penetrance from normal to severe clinical symptoms [14,22].

Furthermore, Xp22.33 is a chromosome region in which CNVs were found. For example, a duplication at Xp22.33/Yp11.32 (chromosome X pseudoautosomal region 1- PAR1), containing *CRLF2* and *CSF2RA* was observed in a patient with autism [23]. Moreover, microduplication at Xp22.33 involving *SHOX* and *SHOX* enhancer elements has been described in patients with ASD and Asperger’s syndrome [24]. 

Here, we describe a patient with severe myoclonic epilepsy, microcephaly, hypertelorism, dysmorphisms as well as severe psychomotor delay and severe intellectual disability (ID) having a microdeletion at the 7q35 region associated with two microduplications at the 15q13.3 and Xp22.33 regions. To our knowledge, the simultaneous presence of these three CNVs in a patient with neurodevelopmental disorders has not been previously reported.

## 2. Materials and Methods

### 2.1. Proband and Family

The proband, an adult female of 39 years old, was born by spontaneous delivery from healthy consanguineous parents (cousins- third-degree relatives). She was born by eutocic delivery after normal pregnancy and was nursed for 24 months. She had her first episode of severe myoclonic epileptic crisis at the age of 7 months. She said her first words at the age of 12 months, and she walked alone at the age of 18 months with slightly flexed knees. At the age of 8 years, she was not able to write or count, and she needed a support teacher for the school. However, at the age of 8 years, she was independent as regards personal care such as dressing and combing her hair.

**Figure 1:**
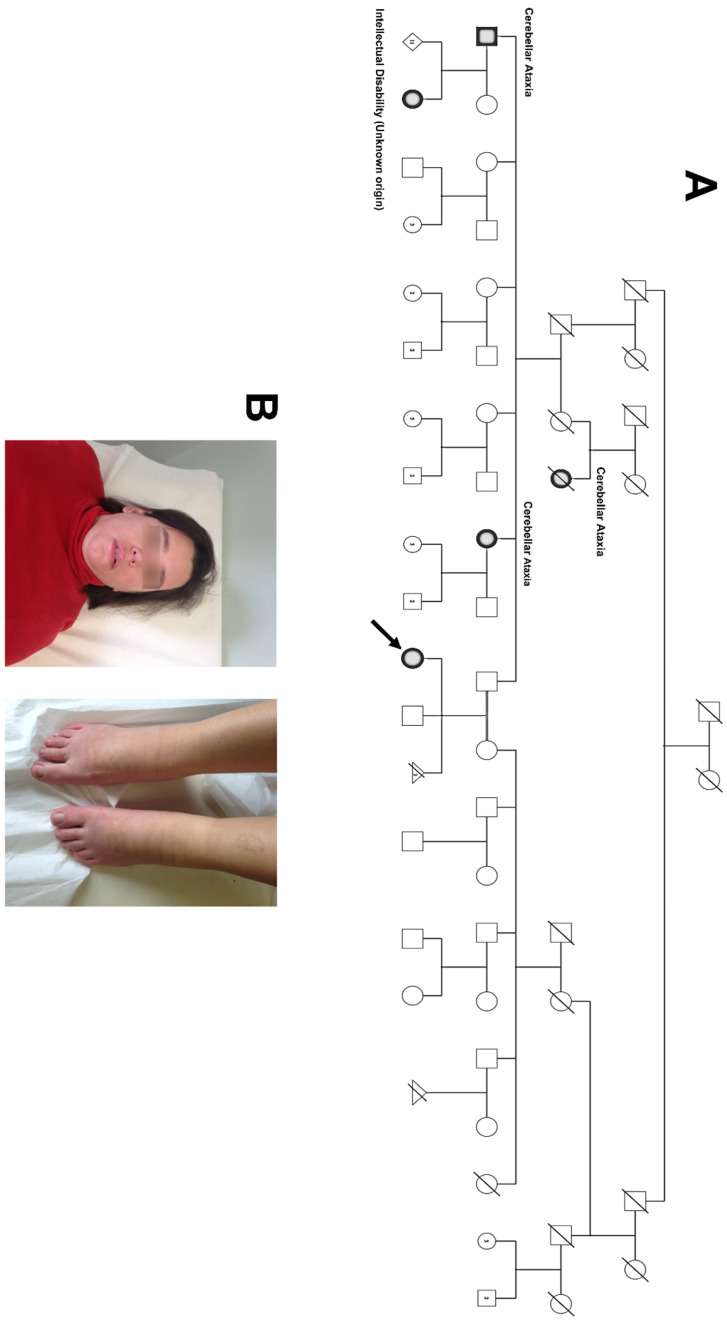
Three-generation family pedigree showing the proband (arrow) and relatives (**A**) and view of the proband phenotype (**B**). The shaded circles represent the affected individuals and the arrow indicates the proband. The numbers inside the circles and squares indicate the number of children.

On physical examination at the age of 36 years, she had a broad forehead with low hairline, sparse eyebrows, abnormal ear position, well-spaced teeth, an ogival palate, microcephaly and hypertelorism (Figure 1B). In addition, the proband had lower limbs dysmetria which was the cause of several postural abnormalities, including the decrease in lumbar lordosis, trunk torsion and the straightening of the neck. Furthermore, she had a lower limb flexor, lumbar hyperlordosis, bilateral coxa valga and a mild coxofemoral osteoporosis (on the left). EEG showed multi-focal spikes whereas encephalon NMR did not reveal morphological brain alterations, with the exception of the sphenoid sinus agenesis. Weight at the age of 36 years was reported at 76 kg (97th centile), and height was 169 cm (75–90th centile).

Proband’s father and brother were healthy, whereas proband’s mother was affected by hypothyroidism (Figure 1A). The proband’s father has two relatives affected by cerebellar ataxia. 

### 2.2. Molecular Analysis

A Chromosomal Microarray Analysis (CMA) was performed on the patient’s DNA extracted from peripheral blood using 4×180 oligo-array platform (Agilent Technologies, Santa Clara, CA, USA). The assay was carried out according to the manufacturer’s instructions, and data were analysed by Agilent CytoGenomics (v. 3.0) software. 

Confirmation and segregation tests on the patient’s and parents’ DNA were performed by Sybr Green qPCR [25] on *CNTNAP2* (7q35), *CHRNA7* (15q13.3) and *CRLF2* (Xp22.33/Yp11.32) genes, using the *TERT* (5p15.33) gene as reference.

### 2.3. Statement of Ethics

Written informed consent was taken for the images present in this case report. The authors declare no ethical conflicts to disclose. This study was approved by the Ethical Committee of “*Regione Calabria, sezione area centro*” (Italy); code: Prot. n. 63; date of the approval: 20 February 2020.

## 3. Results

**Figure 2:**
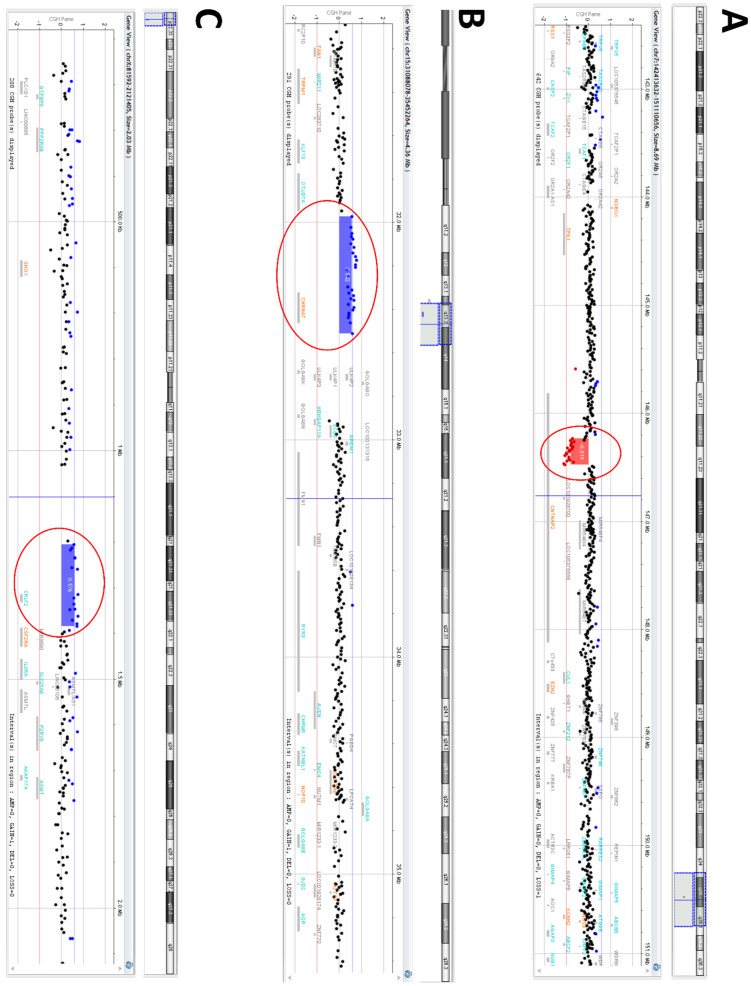
Oligoarray profile of our patient showing three different CNVs: (**A**) 7q35 microdeletion (red bar), involving the exon 2 of *CNTNAP2* gene (NM_014141.6; ENST00000361727.3), (**B**) 15q13.3 microduplication (blue bar), including the entire *CHRNA7* gene, and (**C**) pseudoautosomal region Xp22.33 microduplication (blue bar), encompassing the entire *CRLF2* gene.

CMA of the patient revealed a microdeletion on chromosome 7q35 spanning about 240 kb in size (arr[GRCh37] 7q35(146236230_146475758)×1 pat), involving partially *CNTNAP2* (OMIM *604569) gene (Figure 2A), 15q13.3 microduplication of about 538 kb (arr[GRCh37] 15q13.3(31972646_32510863)x3 mat) (Figure 2B) and microduplication of pseudoautosomal region (PAR1) Xp22.33 of about 178 kb (arr[GRCh37] Xp22.33(1205802_1383843)×3 mat) (Figure 2C), including *CHRNA7* (OMIM *118511) and *CRLF2* (OMIM *300357) genes, respectively. 

**Figure 3:**
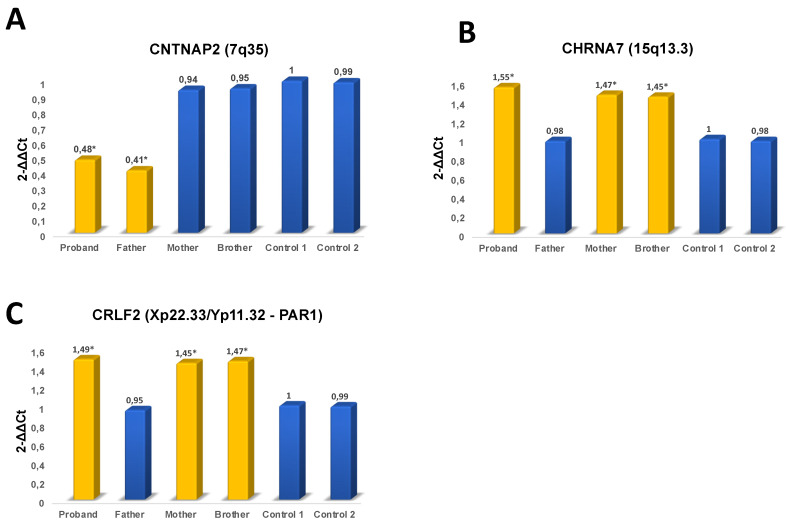
Sybr Green qPCR assay. (**A**) Paternal segregation of 7q35 microdeletion. (**B**) Maternal segregation of 15q13.3 and (**C**) PAR1 microduplications.

Sybr Green qPCR assay confirmed CMA data in our patient and demonstrated the paternal segregation of 7q35 microdeletion, whereas 15q13.3 and PAR1 microduplications were inherited from her mother with hypothyroidism. The healthy brother of the proband was a carrier of 15q13.3 and Xp22.33 microduplications; both inherited from his mother (Figure 3). 

## 4. Discussion

Here, we report on a patient presenting with severe myoclonic epilepsy, microcephaly, hypertelorism, dysmorphisms as well as severe psychomotor delay and intellectual disability. CMA of the proband identified three different CNVs, a paternal 7q35 microdeletion in combination with two maternal microduplications at 15q13.3 and Xp22.33. 

The 7q35 microdeletion encompasses partially the intron 1 and the entire exon 2 of *CNTNAP2*. This gene encodes for a protein of the neurexin superfamily, known as contactin-associated protein 2 (Caspr2), which is a transmembrane protein involved in cell–cell interactions between neurons and glial cells and plays a crucial role in the axonal differentiation and guidance [9]. 

It has been shown that intron 1 of *CNTNAP2* contains two important regulatory regions for the transcription factors Storkhead box 1A (STOX1A) and Forkhead box P1 (FOXP1) [26]. These two regulatory regions inside intron 1 of *CNTNAP2* are essential for the correct control of CNTNAP2 expression; a deletion within intron 1 covering the *FOXP2* binding sites has been observed in patients suffering from language delay and autism [27] and bipolar disorder [28]. A similar deletion at 7q35 encompassing a part of intron1 and exon 2 of *CNTNAP2* in combination with other chromosomal rearrangements has been observed in a patient with speech delay and ASD [29].

Other genetic studies support the link between *CNTNAP2* dysfunction and neurodevelopmental disorders. It has been described a patient with non-specific dysmorphic features, speech problems and learning disability having an intragenic deletion which disrupts the reading frame of the *CNTNAP2* [27,30]. Importantly, it has also been observed a deletion at 7q35 that interrupts the *CNTNAP2* gene in a subject with Alzheimer’s disease (AD) [9]. In addition, DNA deletions disturbing one of the two copies of *CNTNAP2* were observed in certain subjects with epilepsy, autism, bipolar disorder, schizophrenia and ADHD, indicating that *CNTNAP2* was involved in several neurologic or psychiatric diseases [28].

Conflicting results have been described in the literature regarding the role played by *CNTNAP2* in the development of neurological diseases. Toma et al., in their large comprehensive study, showed that multiple classes of DNA variations such as SNPs, de novo variants and CNVs of *CNTNAP2* are unlikely to play a role in the development of psychiatric or neurological disorders [28]. 

In addition, by studying a large family with bipolar disorder, they showed that a CNV deletion that removes the FOXP2 transcription binding site in intron 1 of *CNTNAP2* (chr7:146203548-146334635) is not only present in affected individuals but also in an unaffected relative. Therefore, it was observed that this CNV deletion is unlikely to be highly penetrant and did not segregate with bipolar disorder in the above-described family [28]. The limited role of this CNV deletion in the susceptibility of neurological disorders could explain why the father’s proband in our case report, who is a healthy individual carrying a similar CNV deletion (chr7:146236230-146475758), did not develop a clinical phenotype. In his review, Martin Poot suggests that intragenic *CNTNAP2* deletions may have incomplete penetrance, as a consequence of the variable expression of the *CNTNAP2* allele [27]. 

In addition, by studying an extended family, Toma C. et al. observed that a deletion which removes the FOXP2 binding site in intron 1 of *CNTNAP2* does not segregate with the disease, indicating that heterozygous *CNTNAP2* deletions are not always fully penetrant [28].

The chromosome 15q13 region has been observed to be a hotspot for CNVs because it contains low copy repeat elements (LCRs), which promote non-allelic homologous recombination (NAHR) resulting in chromosomal microduplications and microdeletions [13]. For example, 5q13.3 duplication has been associated with epilepsy [15], ASD [14,17], Tourette’s syndrome (TS) [20], bipolar disorder (BPD) [14,19], intellectual disability (ID) [14], ADHD [16], adult onset schizophrenia (AOS) [21], behavioural disorders [21], and language impairment [17,18]. 

The 15q13.3 microduplication encompasses the *CHRNA7* gene, which codes for α7 nicotinic acetylcholine receptor (α7nAChRs) in the brain [13]. α7nAChRs plays a crucial role in signal transduction at synapses and therefore is important in synaptic plasticity, memory and learning [13].

Although it has been shown that deletions at 15q13.3 in patients with neuropsychiatric phenotypes manifest incomplete penetrance (80%) [31] and much lower penetrance for duplications [19], *CHRNA7* dosage sensitivity was suggested as the cause of several of the neuropsychiatric and neurodevelopmental phenotypes detected in patients harbouring 15q13.3 CNVs [14]. 

15q13.3 duplication have been shown to have incomplete penetrance in two patients with childhood-onset schizophrenia [12]. Furthermore, Cooper GM. et al. in their study showed that 15q13.3 is incompletely penetrant (0.83) [32].

Cytokine receptor-like factor 2 (*CRLF2*) gene mapped at the PAR1 on the short arm of the X and Y chromosomes (Xp22.33/Yp11.32) [33]. CRFL2, also known as thymic stromal lymphopoietin receptor subunit (TSLPR), is a type I cytokine receptor that forms a functional complex with IL-7 receptor α chain (IL-7Rα) and thymic stromal lymphopoietin (TSLP) [34]. The activation of IL-7Rα/TSLPR complex by the cytokine TSLP induces the phosphorylation and activation of the JAK/STAT signalling pathways, resulting in the regulation of the inflammatory Th2 responses, peripheral T cell homeostasis and dendritic cells-mediated central tolerance [35]. It has been shown that the cytokine TSLP, which binds CRLF2, is also expressed by astrocytes in the spinal cord and by choroid plexus epithelial cells in the brain, and that it is upregulated in the myelin-degenerative central nervous system [36]. Currently, the TSLP signalling is not entirely understood; for instance, it is also involved in the stimulation of sensory neurons [37].

Until now, patients with neurodevelopmental phenotypes having microduplications at Xp22.33 involving only *CRFL2* were not yet described. *CRLF2* copy number gain and therefore dysregulation of *CRLF2* expression has been described only in patients with acute lymphoblastic leukaemia (ALL) [38]. Two duplications of ∼807 kb and ∼982 kb at Xp22.33 chromosome that includes *CRLF2* and other genes such as *SHOX*, *CSF2RA* and *IL3RA* associated with abnormality of the nervous system were annotated in the database of chromosomal imbalance and phenotype in humans (DECIPHER n. 289637 and n. 294106). Considering these findings, *CRLF2* dysregulation could affect neuronal signalling and have an additive role in the development of the brain disorders observed in the proband, but further studies are needed to confirm this hypothesis.

To our knowledge, the concomitant presence of these three private CNVs in a patient has not previously been reported.

The girl’s father is a carrier of 7q35 microdeletion, and her mother was found to have microduplication of 15q13.3 and Xp22.33, the same as the proband’s brother, but without neurodevelopmental phenotypes. This suggests that each of the CNVs alone described above are not pathogenic enough to induce clinical manifestations, whereas the simultaneous occurrence of these three CNVs (7q35 microdeletion, 15q13.3 and Xp22.33 microduplications) may induce the brain disorders observed in our patient.

The two-hit model could be an explanation for phenotypic differences between probands and their carrier parents [39,40]. This model hypothesises that an additional hit is required during development to induce a more acute clinical phenotype [41]. 

We do not exclude that microcephaly, severe psychomotor delay and dysmorphic signs observed in the proband could be due to the fact that proband’s parents are third cousins. In support of this hypothesis, Rodenas-Cuadrado P. et al. observed facial dysmorphisms and psychomotor problems in a case report in which the proband’s parents were consanguineous (first degree cousins) carrying a 203 kb deletion encompassing exons 2–3 of the *CNTNAP2* (hg38; Chr7:146.711.006-146.914.175) [42]. 

It is essential to underline that the interpretation of the clinical significance of small CNVs (∼500 kb), such as those observed in this patient, is very challenging because their role in the etiopathogenesis of several neurological seems to be associated to different factors, among which multifactorial effects or epigenetic alteration modifying gene expression [1]. Consequently, this sporadic case with one inherited microdeletion at 7q35 and two inherited microduplications at 15q13.3 and Xp22.33 make genotype-phenotype correlation very difficult. 

The main limitations of this study are the absence of direct sequencing and whole exome/genome sequencing for the proband and her parents. Sanger sequencing could give us important information regarding the identified variants, whereas whole exome/genome sequencing can be used to exclude other genetic factors that can contribute to the observed phenotypes. In addition, since family history showed that proband’s parents are third cousins, it is possible that other genetic factors than those described by us could be involved in the development of the observed phenotype. 

Importantly, future functional studies are needed to better understand the potential role of *CNTNAP2*, *CHRNA7* and *CRLF2* genes in neurodevelopmental disorders.

In conclusion, we hypothesise that the simultaneous presence of these three CNVs has an additive effect on phenotype, and together with other environmental or genetic factors, could be responsible for the clinical manifestations of the affected female described here.
